# Supplementary figures and images for: HIV co-infection is associated with reduced Mycobacterium tuberculosis transmissibility in sub-Saharan Africa
Source: PLoS Pathog. 2024 May 2;20(5):e1011675. doi: 10.1371/journal.ppat.1011675 (PMC11093396; doi:10.1371/journal.ppat.1011675)

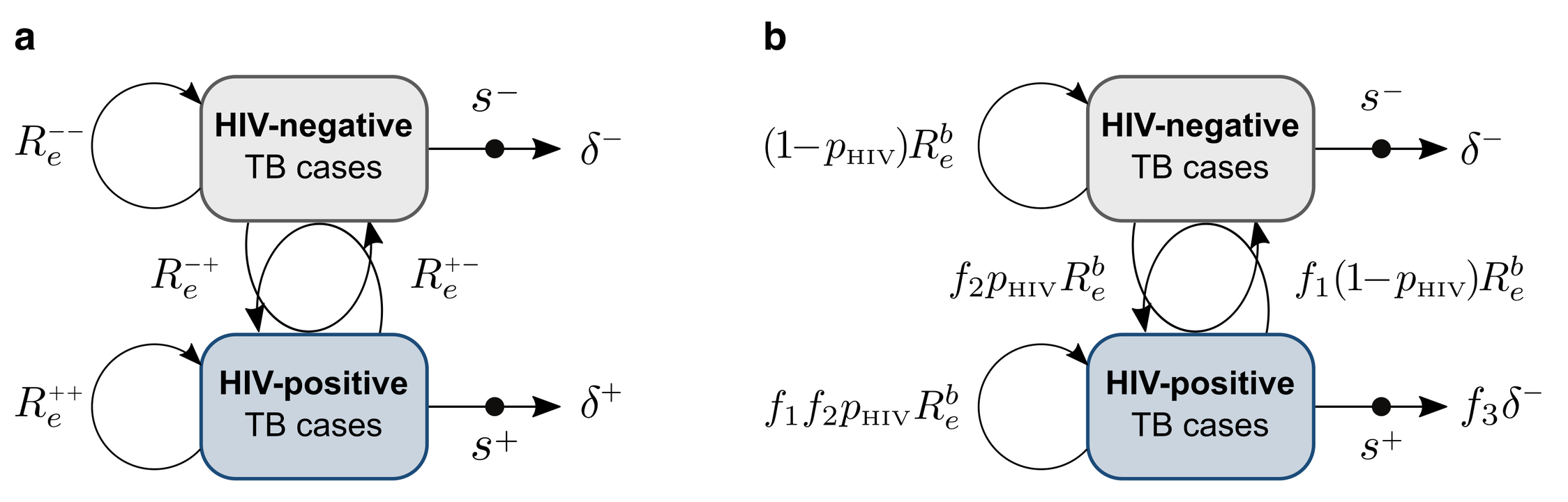

Supplement: S1 Fig — Phylodynamic model used to estimate HIV effects on Mtb transmission, based on a structured birth-death model with HIV-negative and HIV-positive TB cases representing different subpopulations. a) Each subpopulation has its own rate of becoming uninfectious (indicated as δ− and δ+) and sampling rate (indicated as s− and s+). Transmission events occur within each subpopulation with reproductive numbers indicated as Re-- and Re++, and between subpopulations with effective reproductive numbers indicated as Re-+ and Re+-. b) For the analyses in this study, the model was reparametrized by expressing the reproductive numbers as a function of a base Re (Reb), the HIV prevalence in the general population (pHIV), the multiplicative effect of HIV co-infection on the Re of TB patients (f1), the multiplicative effect of HIV co-infection on the risk of developing active TB when exposed (f2), and the multiplicative effect of HIV co-infection on the rate of becoming uninfectious (f3). (TIF) [file ppat.1011675.s001.tif]

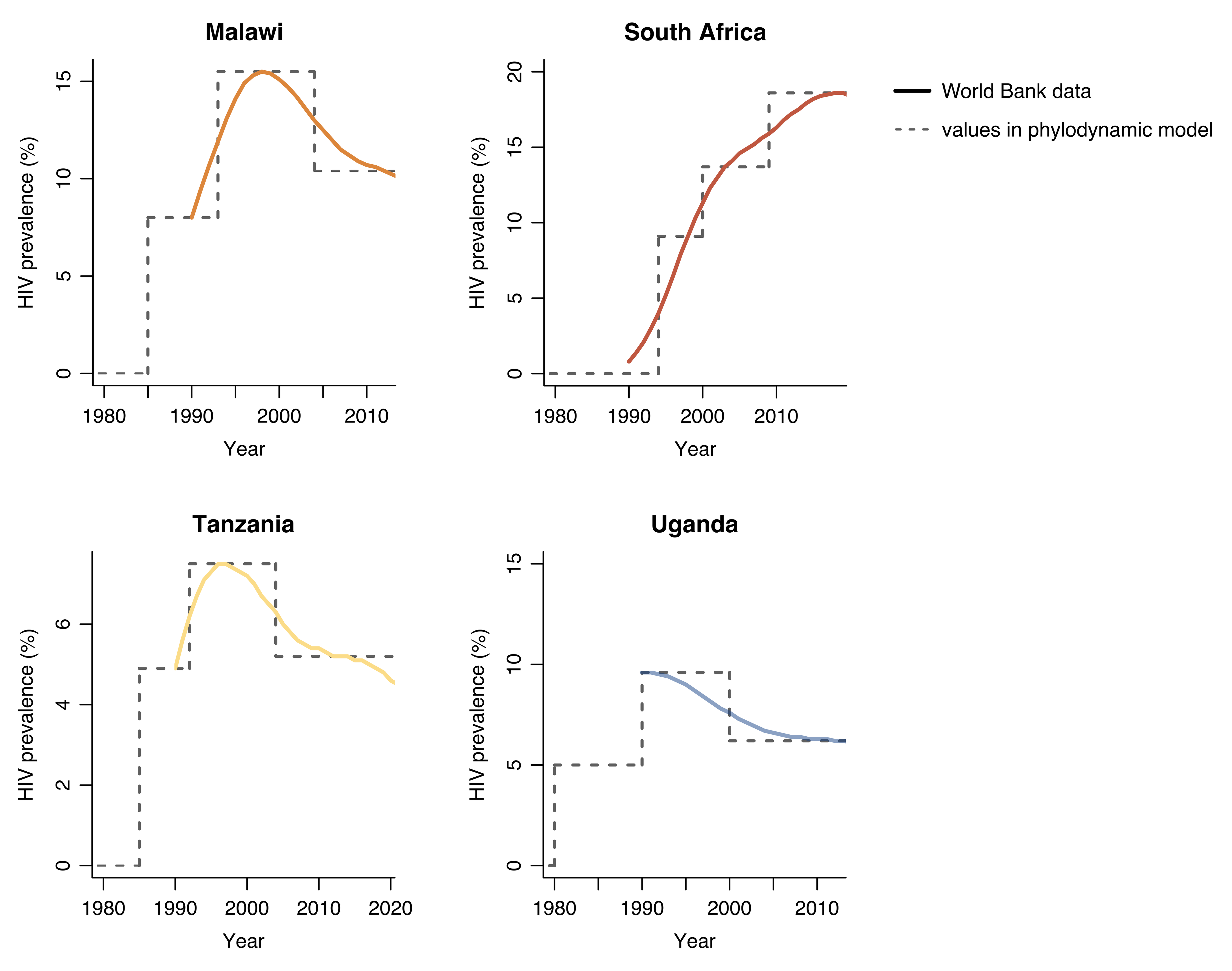

Supplement: S2 Fig — Coloured lines represent the prevalence (% of population ages 15-49) per country over time, as reported by World Bank [44–47], while the dashed grey lines represent the values used in our phylodynamic model. (TIF) [file ppat.1011675.s002.tif]

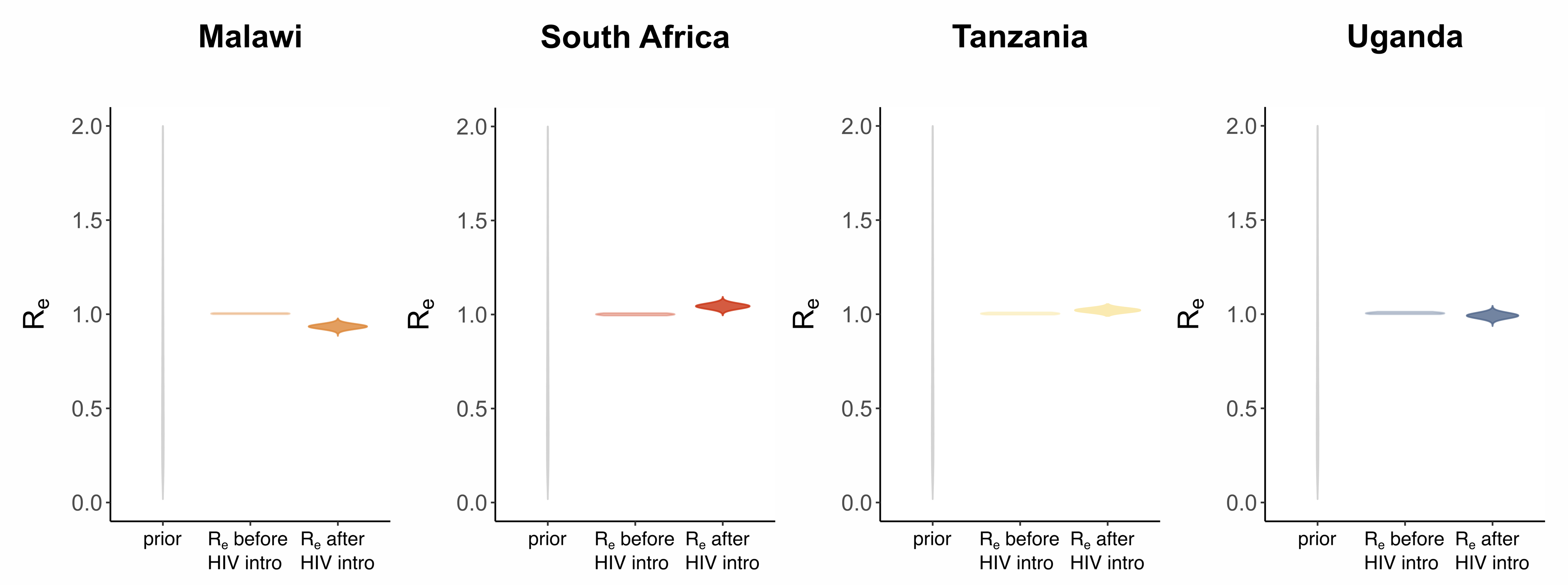

Supplement: S3 Fig — Prior (grey) and posterior (coloured) distributions per sampling location of the estimates of the overall Re before and after the estimated time of HIV introduction into the country, assuming no structure in the population. (TIF) [file ppat.1011675.s003.tif]

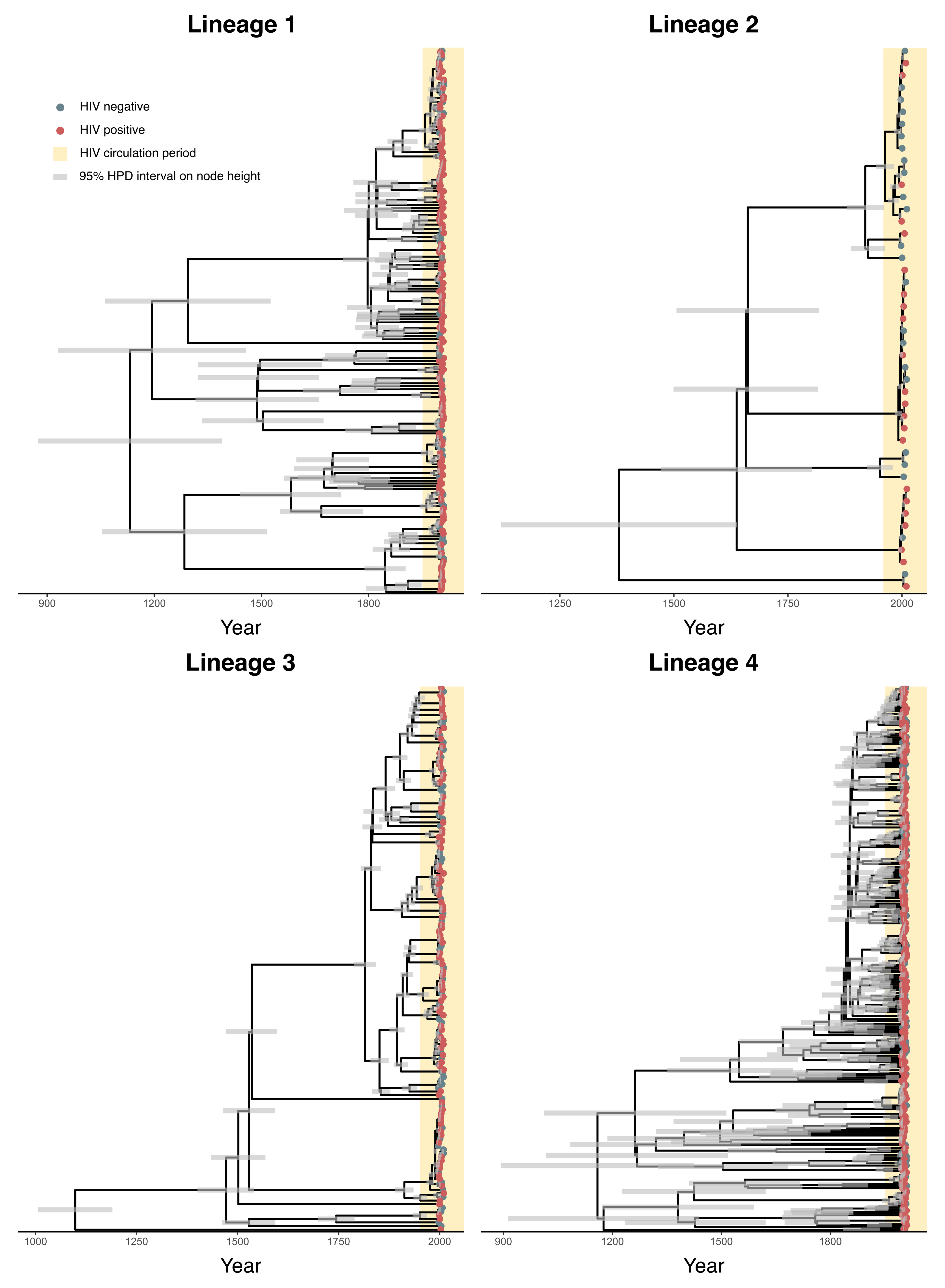

Supplement: S4 Fig — Posterior maximum clade credibility tree per lineage, summarizing the posterior tree distribution resulting from the phylodynamic analyses on the sequences from Malawi, with tips labeled by HIV infection status. (TIF) [file ppat.1011675.s004.tif]

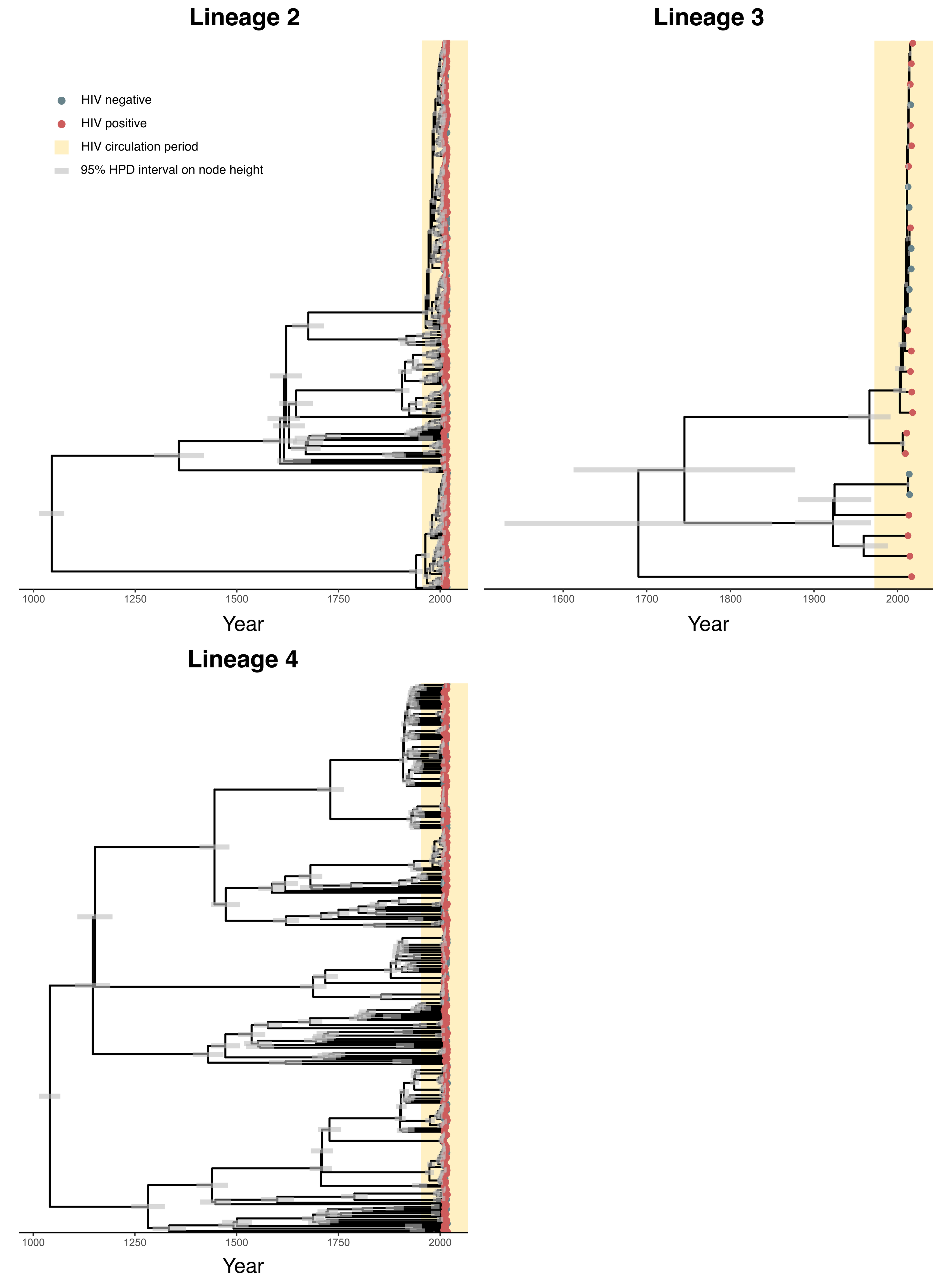

Supplement: S5 Fig — Posterior maximum clade credibility tree per lineage, summarizing the posterior tree distribution resulting from the phylodynamic analyses on the sequences from South Africa, with tips labeled by HIV infection status. (TIF) [file ppat.1011675.s005.tif]

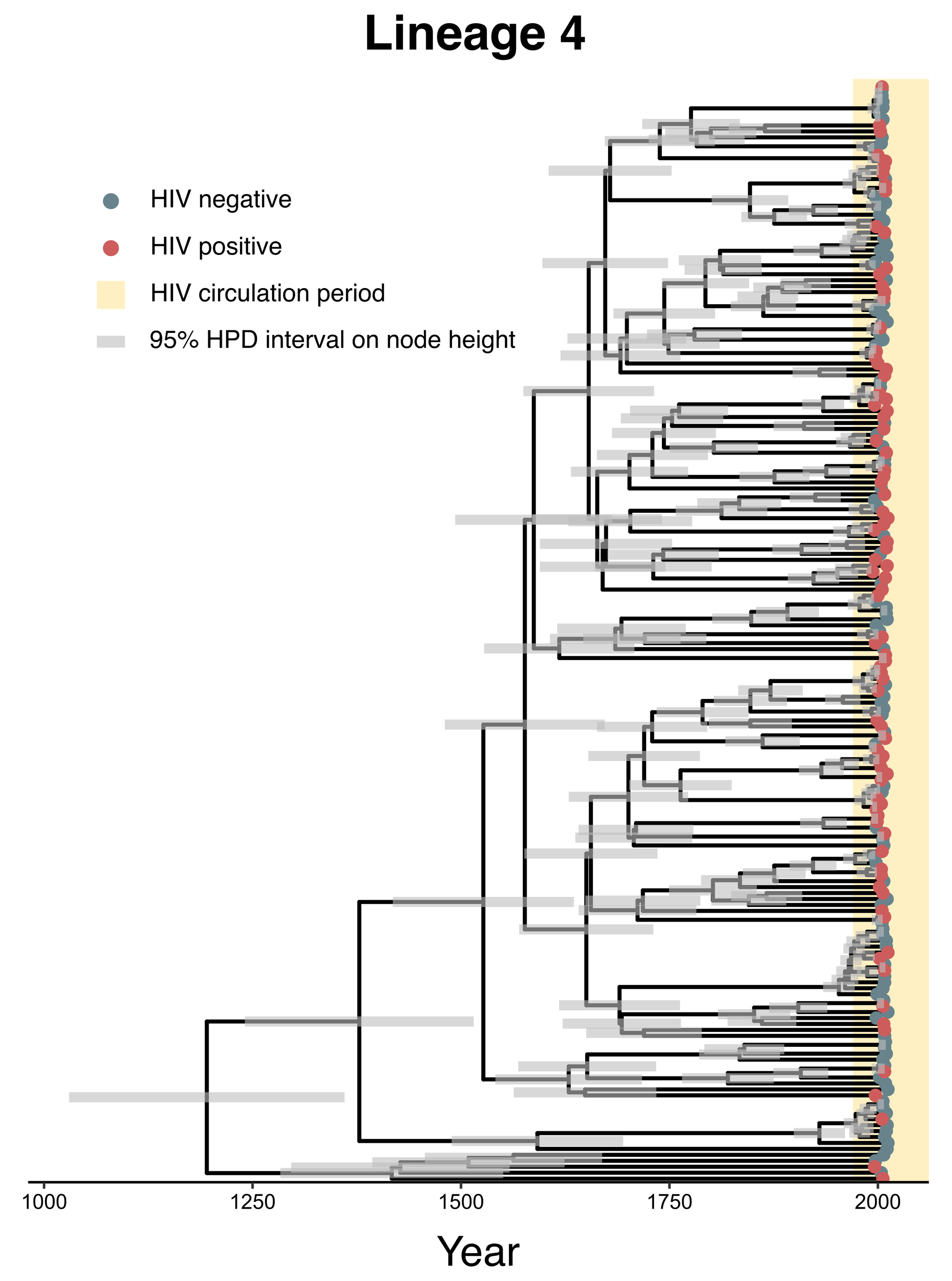

Supplement: S6 Fig — Posterior maximum clade credibility tree, summarizing the posterior tree distribution resulting from the phylodynamic analyses on the sequences from Uganda (lineage 4 only), with tips labeled by HIV infection status. (TIF) [file ppat.1011675.s006.tif]

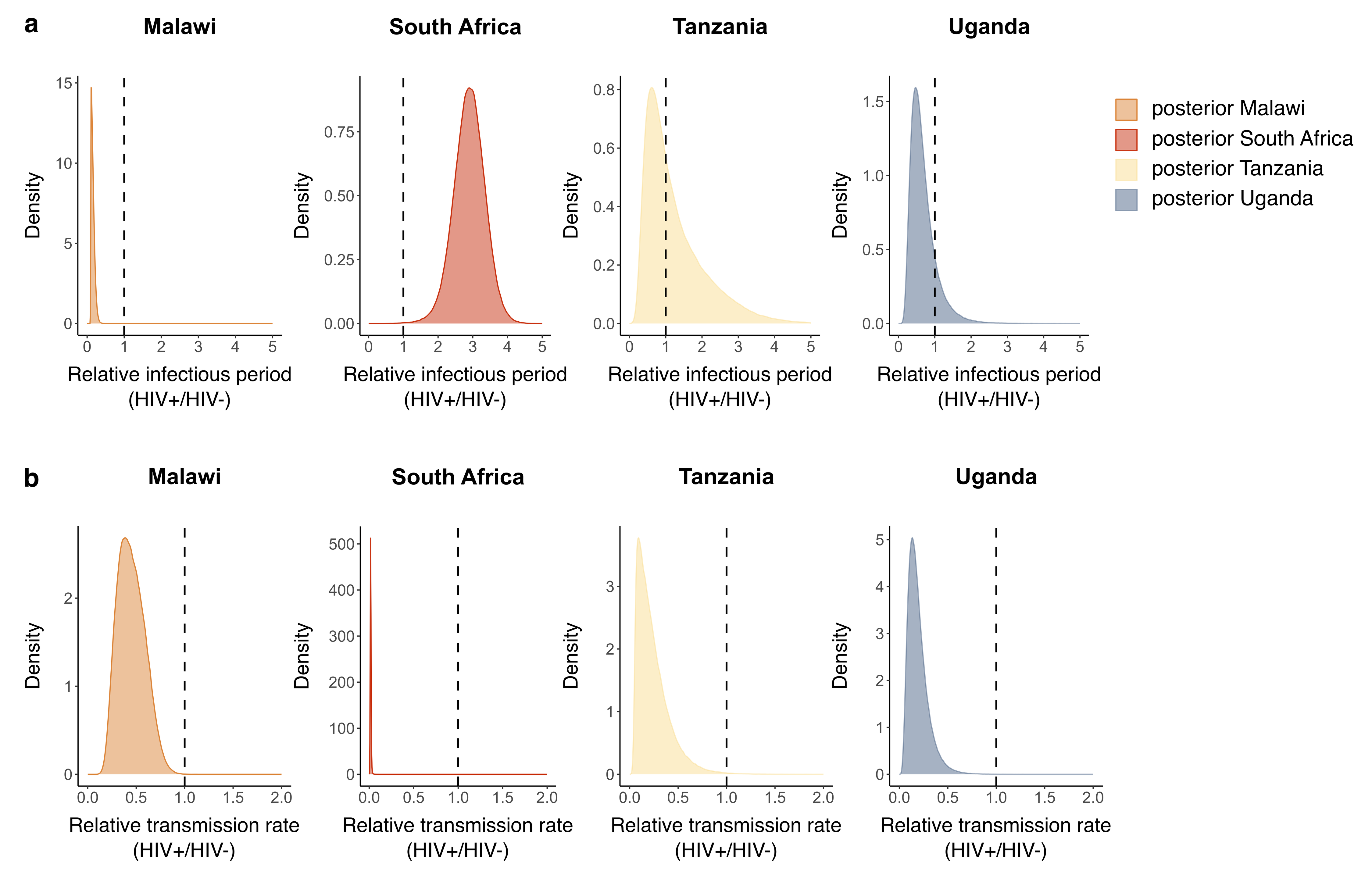

Supplement: S7 Fig — Posterior distributions per sampling location of the estimates for a) the relative Mtb infectious period (HIV-positive relative to HIV-negative individuals), and b) the relative Mtb transmission rate. For all posterior distributions in (b), the 95% HPD intervals do not contain 1. (TIF) [file ppat.1011675.s007.tif]

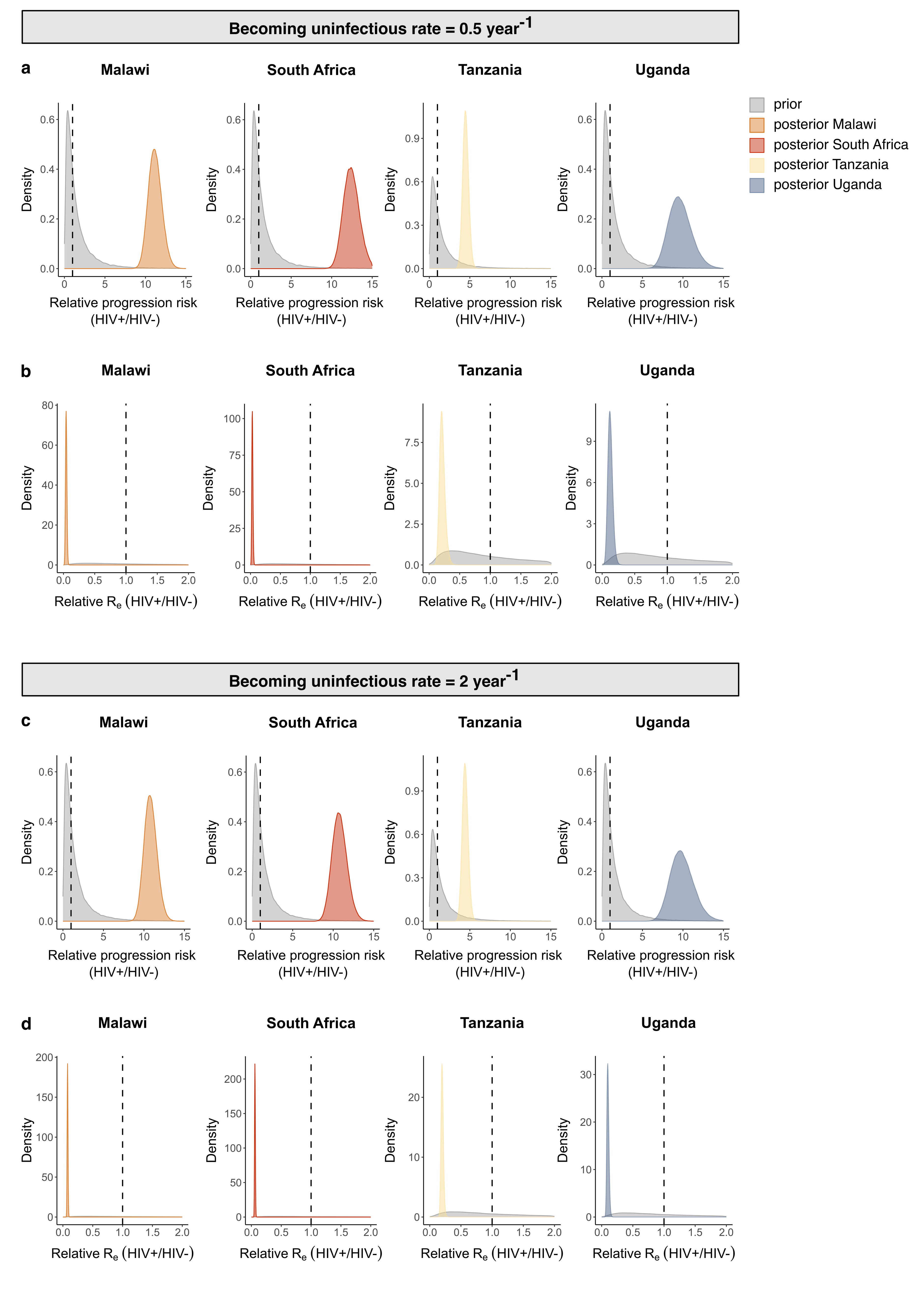

Supplement: S8 Fig — Prior (grey) and posterior (coloured) distributions per sampling location of the estimates for a) the relative risk of developing active TB upon exposure (HIV-positive relative to HIV-negative individuals), assuming a fixed becoming uninfectious rate of 0.5 year−1, b) the relative Re for TB, assuming a fixed becoming uninfectious rate of 0.5 year−1, c) the relative risk of developing active TB upon exposure, assuming a fixed becoming uninfectious rate of 2 year−1, and d) the relative Re for TB, assuming a fixed becoming uninfectious rate of 2 year−1. For all posterior distributions, the 95% HPD intervals do not contain 1. (TIF) [file ppat.1011675.s008.tif]

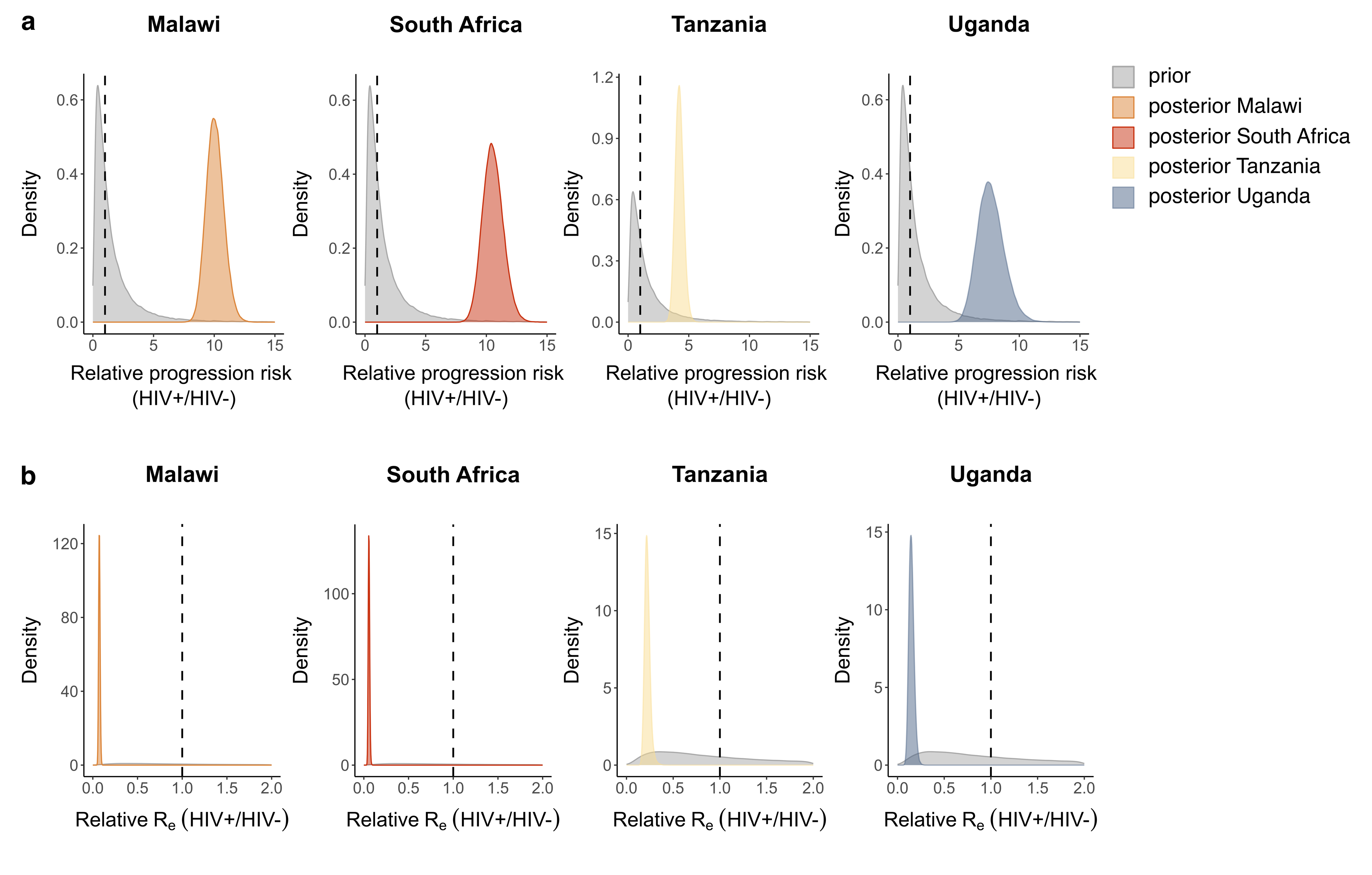

Supplement: S9 Fig — Prior (grey) and posterior (coloured) distributions per sampling location of the estimates for a) the relative risk of developing active TB upon exposure (HIV-positive relative to HIV-negative individuals), and b) the relative Re for TB, assuming Lognormal(0,0.5) priors on the effect of HIV on Mtb transmission (f1) and TB disease progression (f2). For all posterior distributions, the 95% HPD intervals do not contain 1. (TIF) [file ppat.1011675.s009.tif]

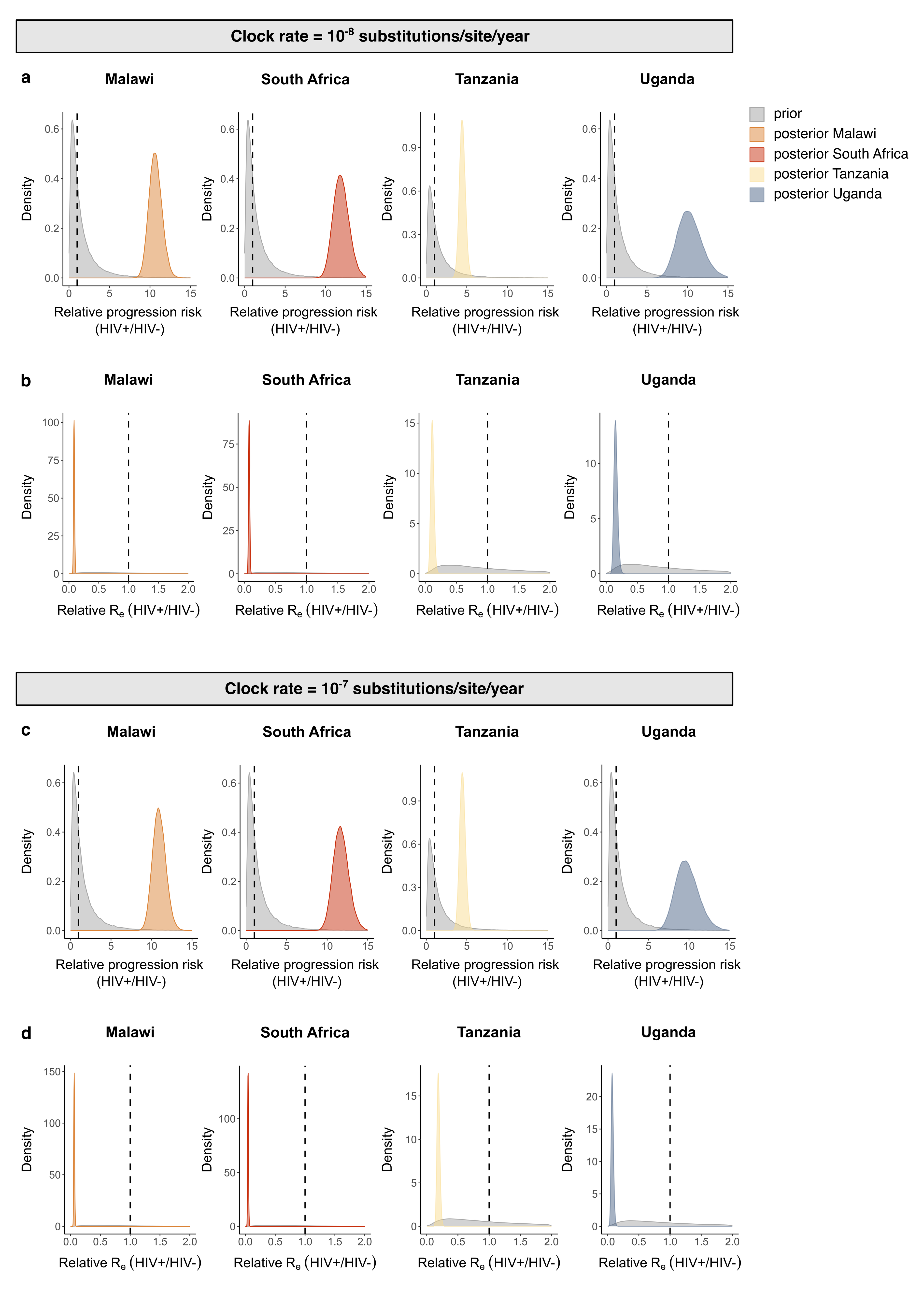

Supplement: S10 Fig — Prior (grey) and posterior (coloured) distributions per sampling location of the estimates for a) the relative risk of developing active TB upon exposure (HIV-positive relative to HIV-negative individuals), assuming a fixed clock rate of 10−8 substitutions per site per year, b) the relative Re for TB, assuming a fixed clock rate of 10−8 substitutions per site per year, c) the relative risk of developing active TB upon exposure, assuming a fixed clock rate of 10−7 substitutions per site per year, and d) the relative Re for TB, assuming a fixed clock rate of 10−7 substitutions per site per year. For all posterior distributions, the 95% HPD intervals do not contain 1. (TIF) [file ppat.1011675.s010.tif]

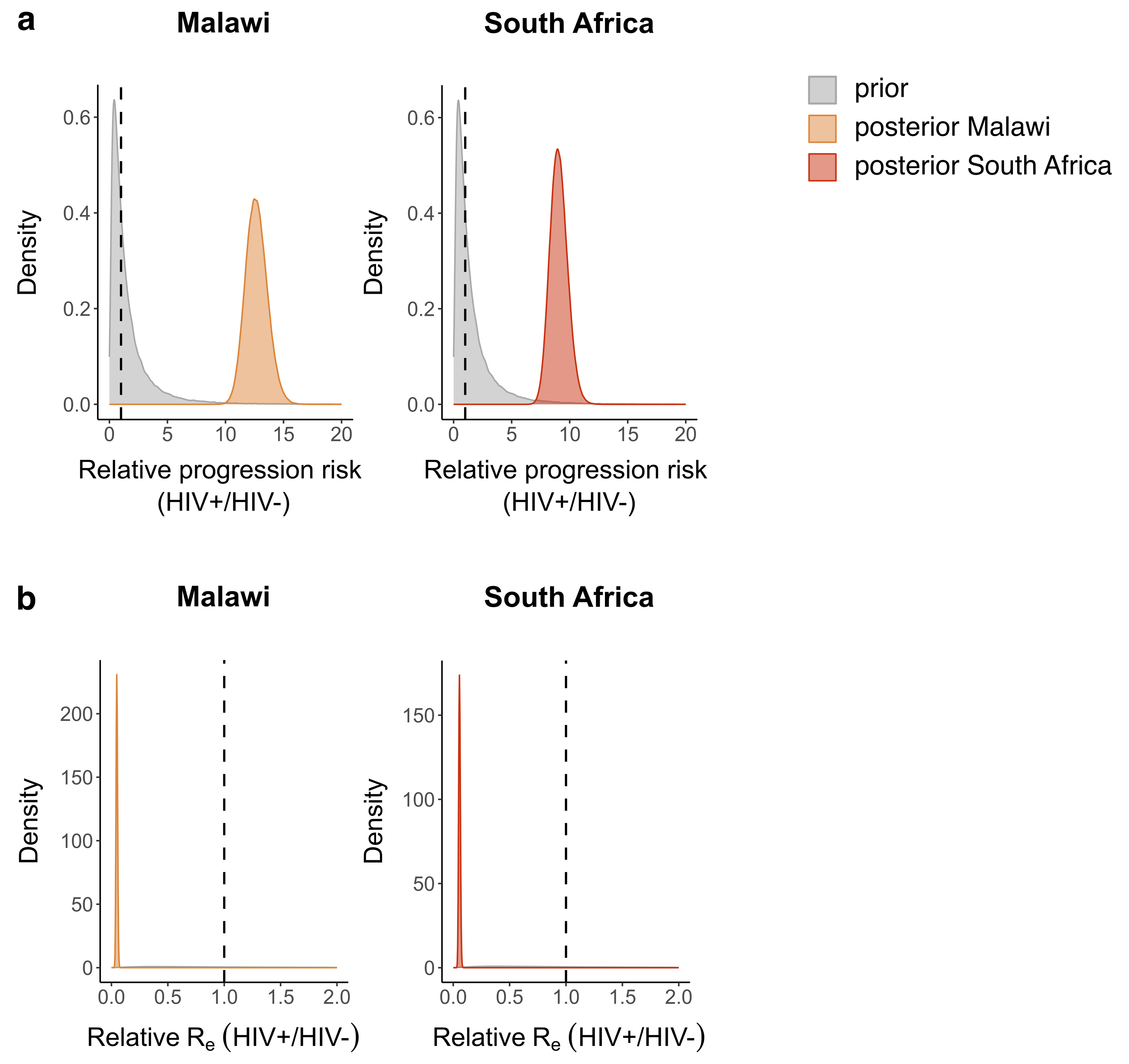

Supplement: S11 Fig — Prior (grey) and posterior (coloured) distributions per sampling location of the estimates for a) the relative risk of developing active TB upon exposure (HIV-positive relative to HIV-negative individuals), and b) the relative Re for TB, using local estimates of the HIV prevalence in Karonga (Malawi) and Khayelitsha (South Africa) (see Materials and methods). For all posterior distributions, the 95% HPD intervals do not contain 1. (TIF) [file ppat.1011675.s011.tif]

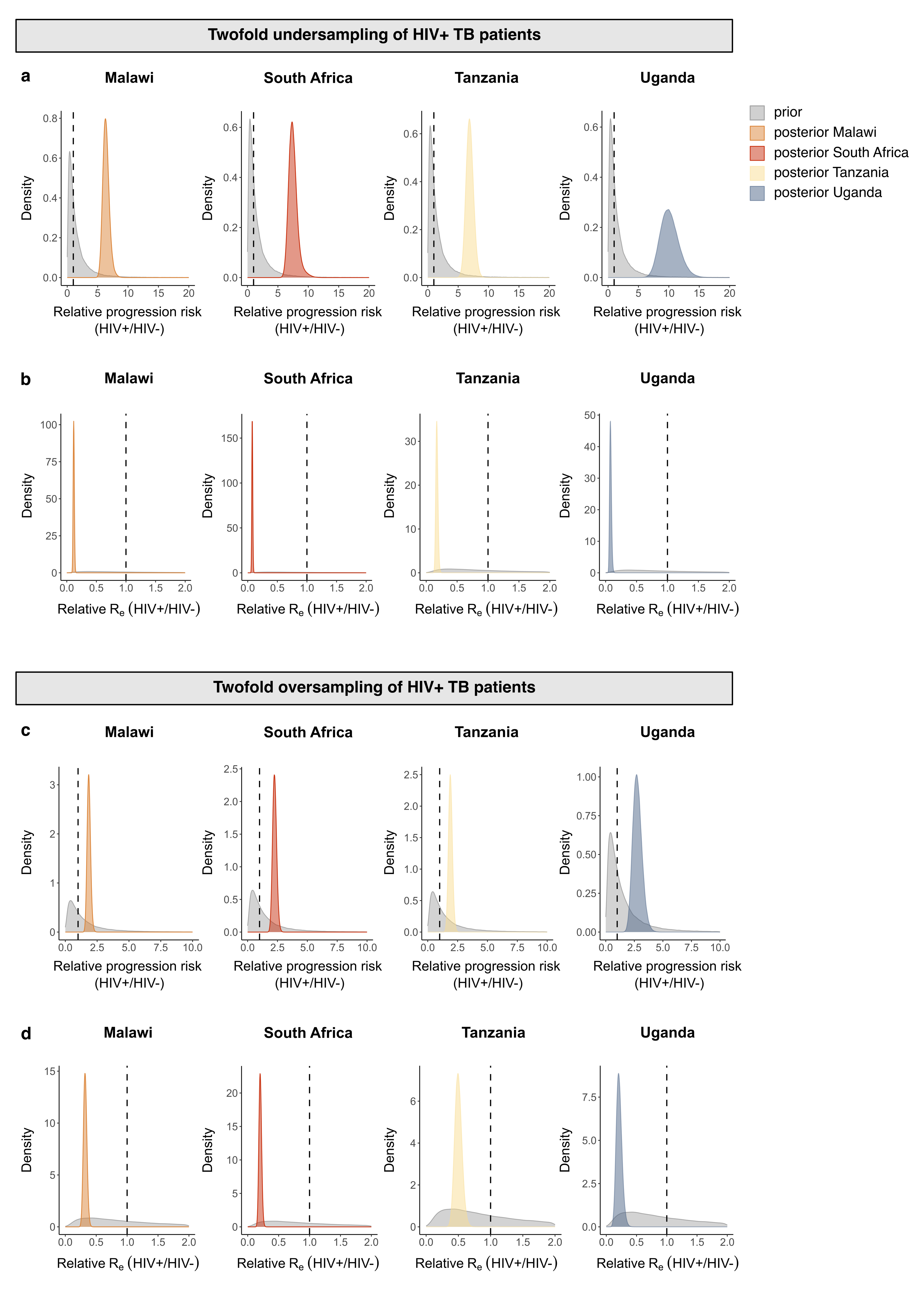

Supplement: S12 Fig — Prior (grey) and posterior (coloured) distributions per sampling location of the estimates for a) the relative risk of developing active TB upon exposure (HIV-positive relative to HIV-negative individuals), assuming a twofold undersampling of HIV-positive compared to HIV-negative cases, b) the relative Re for TB, assuming a twofold undersampling of HIV-positive compared to HIV-negative cases, c) the relative risk of developing active TB upon exposure, assuming a twofold oversampling of HIV-positive compared to HIV-negative cases, and d) the relative Re for TB, assuming a twofold oversampling of HIV-positive compared to HIV-negative cases. For all posterior distributions, the 95% HPD intervals do not contain 1. (TIF) [file ppat.1011675.s012.tif]

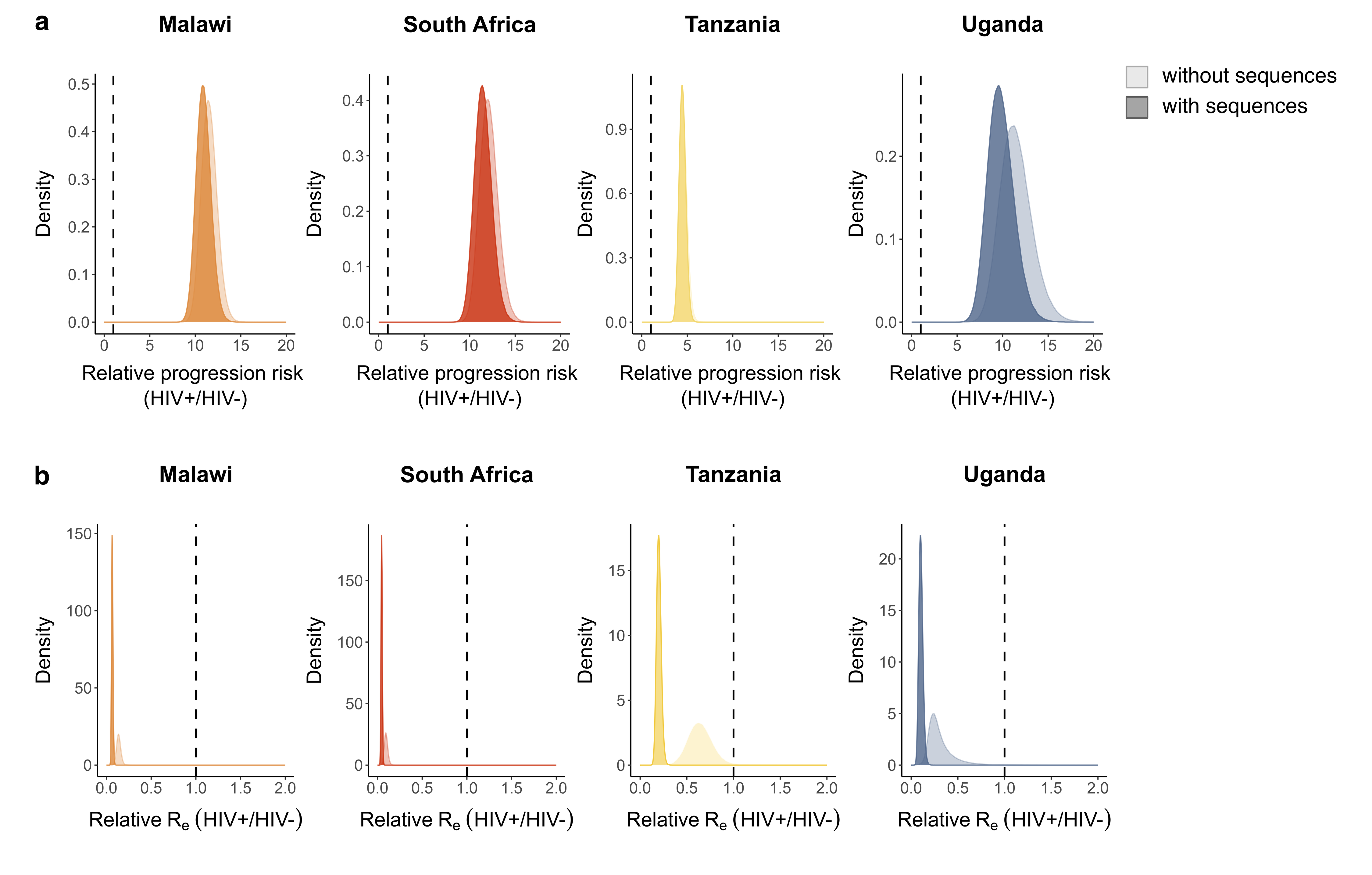

Supplement: S13 Fig — a) Posterior distributions per sampling location of the estimates for the relative risk of developing active TB upon exposure (HIV-positive relative to HIV-negative individuals), only based on the sampling dates and HIV infection status (light colours), or also including the sequences (dark colours). b) Posterior distributions per sampling location of the estimates for the relative Re for TB, only based on the sampling dates and HIV infection status (light colours), or also including the sequences (dark colours). (TIF) [file ppat.1011675.s013.tif]

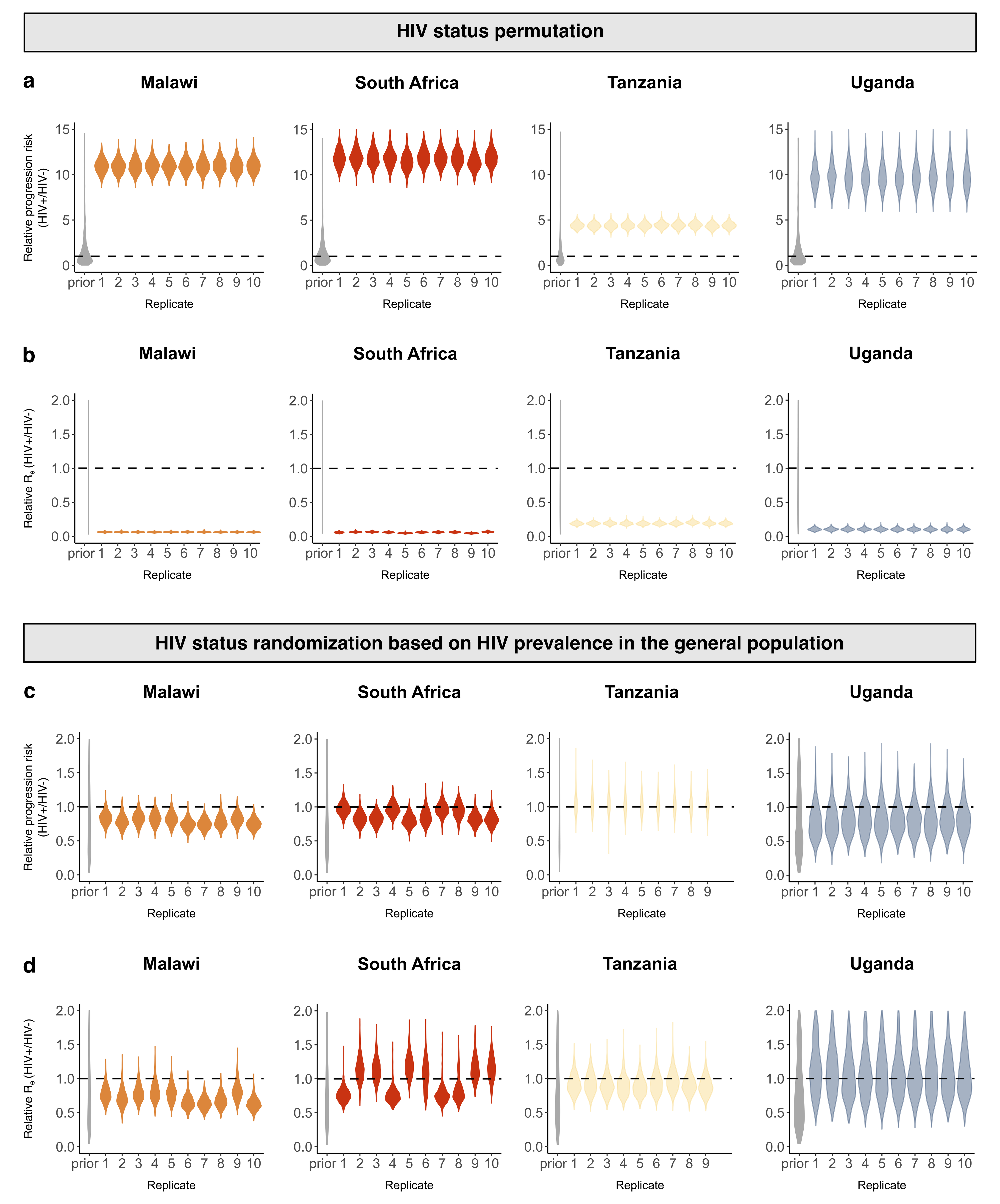

Supplement: S14 Fig — Prior (grey) and posterior (coloured) distributions per sampling location of the estimates for a) the relative risk of developing active TB upon exposure (HIV-positive relative to HIV-negative individuals), on 10 different datasets where the HIV status labels of the patients were permuted, b) the relative Re for TB, on 10 different datasets where the HIV status labels of the patients were permuted, c) the relative risk of developing active TB upon exposure, on 10 different datasets where the HIV status labels were randomly assigned using the average HIV frequency in the general population during the sampling period, d) the relative Re for TB, on 10 different datasets where the HIV status labels were randomly assigned using the average HIV frequency in the general population during the sampling period. (TIF) [file ppat.1011675.s014.tif]

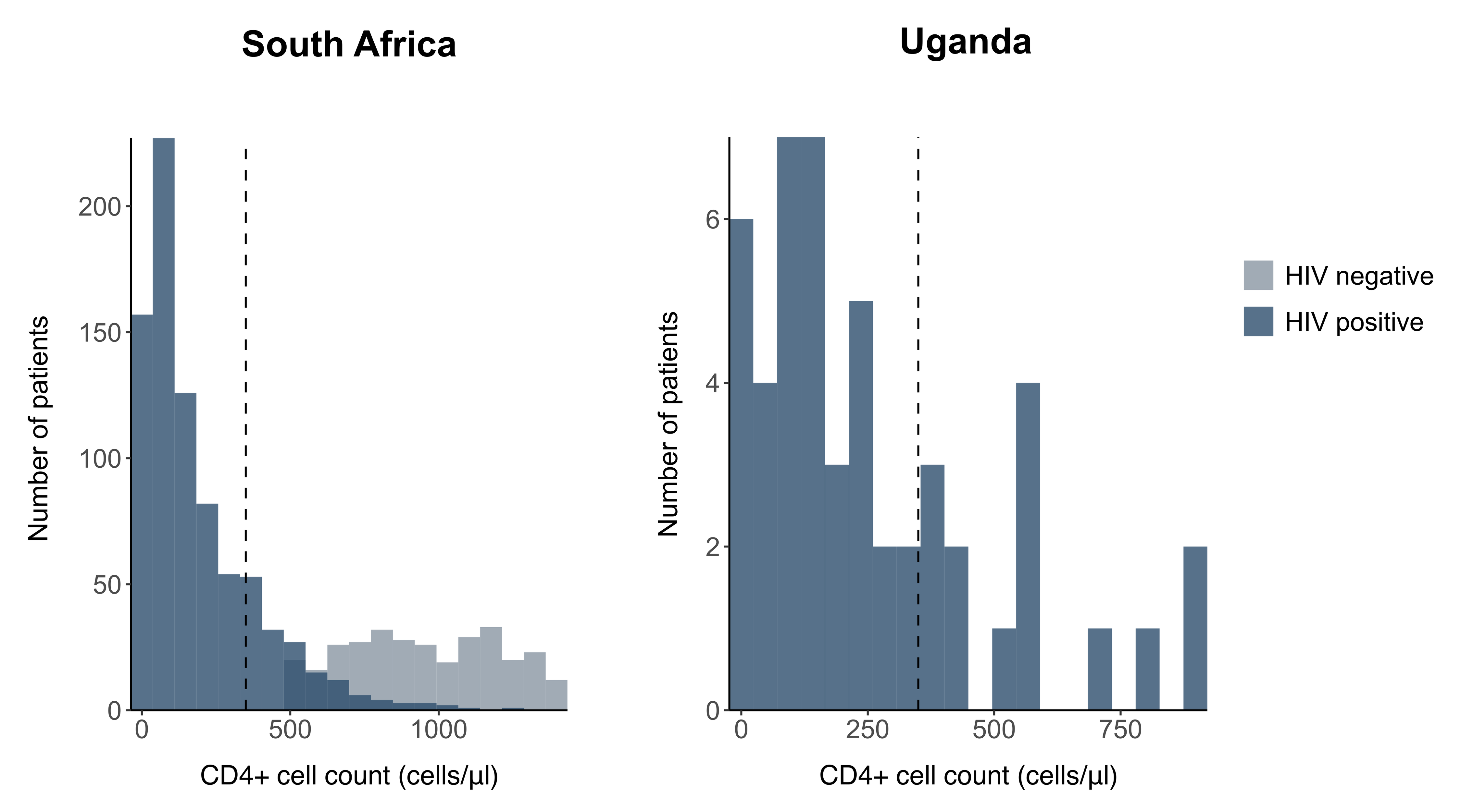

Supplement: S15 Fig — In Uganda, CD4+ T-cell counts were only recorded for HIV-positive patients. The dashed line represents the threshold (350 cells/μl) recommended by WHO to prioritize patients for ART [70]. This threshold was used to stratify the TB patient population. (TIF) [file ppat.1011675.s015.tif]
