# Supplementary material for: HIV co-infection is associated with reduced Mycobacterium tuberculosis transmissibility in sub-Saharan Africa
Source: PLoS Pathog. 2024 May 2;20(5):e1011675. doi: 10.1371/journal.ppat.1011675 (PMC11093396; doi:10.1371/journal.ppat.1011675)
Supplement: S1 Table — (PDF) [file ppat.1011675.s016.pdf]

S1 Table. Observed lineage distribution at the different sampling locations, based on the number of sequences in the datasets. From the Kampala dataset, only lineage 4 isolates were sequenced. For the other datasets, we assumed no lineage bias in sampling or sequencing. Lineages for which more than 400 sequences were availabe were downsampled to 400 for computational feasibility of the phylodynamic analyses.

	Lineage 1	Lineage 2	Lineage 3	Lineage 4
Karonga (Malawi)	196	45	150	818
Cape Town (South Africa)	0	766	27	340
Dar es Salaam (Tanzania)	149	84	502	339
Kampala (Uganda)	0	0	0	185
