# Supplementary material for: HIV co-infection is associated with reduced Mycobacterium tuberculosis transmissibility in sub-Saharan Africa
Source: PLoS Pathog. 2024 May 2;20(5):e1011675. doi: 10.1371/journal.ppat.1011675 (PMC11093396; doi:10.1371/journal.ppat.1011675)
Supplement: S2 Table — (PDF) [file ppat.1011675.s017.pdf]

S2 Table. Prior distributions for the parameters of the multitype birth-death model (see Materials and methods for more information about the individual parameters).

Parameter	Symbol	Prior
Base reproductive number for TB, in a purely HIV-negative population	R_e^b	Lognormal(0,1)
Rate of becoming *Mtb* uninfectious (HIV-negative individuals)	δ^-	1 (fixed)
Multiplicative effect of HIV on *Mtb* transmission	f_1	Lognormal(0,1)
Multiplicative effect of HIV on risk of TB disease development	f_2	Lognormal(0,1)
Multiplicative effect of HIV on rate of becoming *Mtb* uninfectious	f_3	Lognormal(0,0.5)
HIV prevalence	p_{HIV}	fixed (S2 Fig.)
Migration rates		0 (fixed)
Sampling proportion	s	Uniform(0, $\frac{\#\text{sequences}}{\#\text{reported cases}}$)
Probability of removal upon sampling	r	Uniform(0,1)
Clock rate		Lognormal(-16,1)
Time of origin		Uniform(0,1000)
Gamma shape		Exp(1)
A→C substitution rate		Gamma(0.05,10)
A→G substitution rate		Gamma(0.05,20)
A→T substitution rate		Gamma(0.05,10)
C→G substitution rate		Gamma(0.05,10)
G→T substitution rate		Gamma(0.05,10)
