# Supplementary material for: HIV co-infection is associated with reduced Mycobacterium tuberculosis transmissibility in sub-Saharan Africa
Source: PLoS Pathog. 2024 May 2;20(5):e1011675. doi: 10.1371/journal.ppat.1011675 (PMC11093396; doi:10.1371/journal.ppat.1011675)
Supplement: S3 Table — (PDF) [file ppat.1011675.s018.pdf]

S3 Table. Mean and 95 % HPD interval for the clock rate estimates ($\times 10^{-7}$ substitutions per site per year) resulting from the main phylodynamic analyses.

	Lineage 1	Lineage 2	Lineage 3	Lineage 4
Malawi	1.62 [0.98,3.59]	0.65 [0.38,0.95]	0.73 [0.60,1.02]	0.99 [0.66,1.84]
South Africa	-	0.54 [0.50,0.58]	1.37 [0.74,2.05]	0.78 [0.74,0.82]
Tanzania	1.69 [1.17,2.80]	1.03 [0.62,1.45]	0.89 [0.73,1.09]	1.00 [0.88,1.14]
Uganda	-	-	-	0.40 [0.32,0.51]
