# Supplementary material for: HIV co-infection is associated with reduced Mycobacterium tuberculosis transmissibility in sub-Saharan Africa
Source: PLoS Pathog. 2024 May 2;20(5):e1011675. doi: 10.1371/journal.ppat.1011675 (PMC11093396; doi:10.1371/journal.ppat.1011675)
Supplement: S4 Table — (PDF) [file ppat.1011675.s019.pdf]

S4 Table. Total number of reported cases during the sampling period and total number of sequences included in the analyses (after downsampling) at the different sampling locations. In Cape Town, these numbers only apply to RR/MDR-TB cases.

	Reported cases	Sequences
Karonga (Malawi)	4,748	791
Cape Town (South Africa)	3,200	767
Dar es Salaam (Tanzania)	23,964	972
Kampala (Uganda)	15,411	185
