# Supplementary material for: HIV co-infection is associated with reduced Mycobacterium tuberculosis transmissibility in sub-Saharan Africa
Source: PLoS Pathog. 2024 May 2;20(5):e1011675. doi: 10.1371/journal.ppat.1011675 (PMC11093396; doi:10.1371/journal.ppat.1011675)
Supplement: Attachment — Submitted filename: Windelsetal2024_rebuttal.pdf [file ppat.1011675.s021.pdf]

Part I - Summary
Please use this section to discuss strengths/weaknesses of study, novelty/significance, general execution and scholarship.

Reviewer #1: The manuscript uses phylodynamic data from four sub-Saharan African countries to estimate the relative infectiousness of Mtb in people living with HIV (PLHIV) with TB compared to HIV- people with TB. They show that 1) PLHIV are at higher risk of TB disease, and that 2) HIV coinfection is associated with a lower effective reproduction number. These findings are in line with previous studies. They also 3) attempt to determine the extent to which reductions in the reproduction number are due to lower durations of infectious vs lower transmission rates.

There is already a very strong evidence base for 1), with numerous studies providing estimates of RR generated using more direct and reliable methods, and systematic reviews summarising available evidence on risk by CD4 count (https://www.ncbi.nlm.nih.gov/pmc/articles/PMC5733368/) and ART (https://bmcinfectdis.biomedcentral.com/articles/10.1186/s12879-023-08533-0) This study therefore contributes little to scientific knowledge with 1).
There is a much weaker evidence base for 2, and therefore this study has the potential to make more valuable contributions to the literature. I have a number of concerns however, detailed below.

We agree with the reviewer that 1) is not a novel finding of our study. However, we see it serving as an internal positive control, showing that our method independently replicates results that have been generated through other, more direct measurements. We have rephrased some sentences in the manuscript to clarify this:

Lines 104-108: “These results suggest that HIV-positive individuals have a 4- to 11-fold increased risk of developing active TB upon exposure, which is in accordance with the increased TB incidence rate observed in HIV patients [9, 10, 12, 13], and can thus be considered as a 'positive control' in our analyses.”

Lines 185-188: “Our phylodynamic analyses support previous studies showing that HIV/*Mtb* co-infected individuals are at high risk of developing active TB disease compared to HIV-negative *Mtb*-infected individuals. This observation serves as a ‘positive control’ that supports the validity of our method.”

Lines 192-193: “The finding from this and previous studies that HIV co-infection is a strong risk factor for developing active TB disease upon exposure...”

We have also cited the suggested systematic reviews in the Introduction and Discussion.

I find 3), the attempt to distinguish between infectious duration and relative transmission risk, very interesting, and reliable estimates of the contributions of the two factors would be valuable. I do not understand what it is in the model/data that allows the two to be distinguished however, and so cannot judge whether the results are likely to be reliable. I would greatly appreciate more discussion of the data and assumptions that go into distinguishing the two. (Also, see below for concerns around the South Africa estimates).

Both parameters are estimated from the branching patterns observed in the phylogenetic tree, assuming a birth-death model as underlying tree-generating process. In the context of epidemiology, new branching events are assumed to occur at transmission (resulting in a newly infected individual) and branches terminate when infected individuals stop being infectious. The rates of branch generation and extinction correspond to the ‘transmission rate’ and ‘patient recovery rate’ during an epidemic, with the infectious period corresponding to the inverse of the recovery rate.

For an extensive explanation on birth-death models and their mathematical underpinnings, we would like to refer the reviewer to existing literature, including:

- Kendall, D. G. (1948). On the generalized "birth-and-death" process. The Annals of Mathematical Statistics, 19(1):1-15.

- Stadler, T. (2009). On incomplete sampling under birth-death models and connections to the sampling-based coalescent. Journal of Theoretical Biology, 261(1):58-66.
- Stadler, T. (2010). Sampling-through-time in birth-death trees. Journal of Theoretical Biology, 267(3):396-404.
- Kühnert, D., Stadler, T., Vaughan, T.G., Drummond, A.J. (2016). Phylodynamics with migration: A computational framework to quantify population structure from genomic data. Molecular Biology and Evolution, 33(8):2102-2116.

Some relevant model assumptions include:

- Exponentially distributed waiting times until infection, recovery, and sampling events
- Perfect mixing within and between subpopulations (in this case, HIV-positive and HIV-negative subpopulations)

Other model assumptions that are relevant to this study are mentioned in the Discussion (Lines 245-266). The prior distributions on each of the estimated parameters are listed in Table S2.

As also stated in the manuscript, the estimates of the infectious period are highly uncertain and show conflicting results for the different countries, precluding general conclusions about the effect of HIV on the duration of infectiousness. This is not surprising, since the infectious period highly depends on the healthcare setting (access to treatment, treatment efficacy, etc.). However, our results show that, even in very different settings with potentially different relative infectious periods, the relative reproductive number for TB is consistently estimated to be lower than 1, which we consider the main contribution of this study.

Reviewer #2: This manuscript models the effective reproductive number of TB among HIV and non-HIV risk groups in four different Sub-saharan african countries using a phylodynamic approach. There are many assumptions made to allow for this modeling with the advantages of integrating epidmiological, and genetic evolutionary parameters including a strict molecular clock, assumptions around population size changes over time, and the observed sampling dates of cases that were sequenced. This approach doesnot take into account individual or host risk factors, social network differences between people living HIV and those without HIV with regards to TB risk. The study also doesnot consider sampling differences for the patients and their samples that underwent whole genome sequences in the four different countries. One of the studies that they relied on Mtb WGS from South AFrica only sequenced drug resistant isolates. I believe that the authors have done an overall good job of listing limitations and assumptions of their approach, and I do believe they make a valuable contribution to the literature on the topic. I have a few comments that I hoped they could address:

We agree that our approach involves many model assumptions, and we have attempted to carefully verify each of these assumptions through many sensitivity analyses and randomization procedures. The assumptions regarding sampling are tailored to each country specifically (Table S4). Since we agree that there could also be HIV-specific sampling biases, we have now run additional sensitivity analyses showing that these potential biases do not affect the overall conclusions from our study (see below).

As suggested by the reviewer and also mentioned in the Discussion (Lines 237-239), the observed effects on TB transmission can be confounded by various factors, including social factors. As in every transmission study, controlling for these potential confounders is very challenging. However, we report here an observed association between HIV infection and reduced infectiousness for TB, without directly pinpointing the underlying cause of this association (i.e., reduced contacts vs. reduced transmission risk per contact). Nevertheless, the fact that our results are consistent across different locations with potentially highly distinct social network characteristics and other host and bacterial factors (including drug resistance), hints at an intrinsic biological effect of HIV infection on TB transmission.

Part II – Major Issues: Key Experiments Required for Acceptance

Please use this section to detail the key new experiments or modifications of existing experiments that should be absolutelyrequired to validate study conclusions.

Generally, there should be no more than 3 such required experiments or major modifications for a "Major Revision" recommendation. If more than 3 experiments are necessary to validate the study conclusions, then you are encouraged to recommend "Reject".

Reviewer #1: The genomic data came from specific study locations, however country-level HIV prevalence estimates were used. HIV prevalence can vary greatly by region, but also within regions. For instance, HIV prevalence is fairly low in Western Cape compared to South Africa as a whole, however it is likely to be higher in Khayelitsha than in the province as a while. Estimates for the study community should be available for Karonga at least, and potentially for Khayelitsha. The analyses should be repeated using the best available local (and adult only) HIV prevalence estimates.

We initially used country-wide HIV prevalence estimates because these were more accessible than local estimates, but we agree that local estimates would provide a more accurate picture. We now obtained local HIV prevalence estimates for Karonga and Khayelitsha, and have re-run the analyses with these new values. Details of the local prevalence estimates are provided in the Materials and Methods section (Lines 410-418), and the results of the analyses are included as Supplementary Figure S11. While the resulting HIV effect estimates are slightly shifted compared to the original results, the qualitative conclusions remain unchanged.

We did not manage to get accurate, timely estimates for Temeke and Kampala. However, since our qualitative conclusions are consistent across all countries (with variable HIV prevalence) and when applying local estimates for Malawi and South Africa, we anticipate that the local variability within Tanzania and Uganda is unlikely to affect the overall conclusions of our study.

ART reduces the risk of developing TB, and it is highly probable that it also increases TB infectiousness. Country-level ART coverage varied from 14% to 70% in South Africa, from 0% to 34% in Uganda, 0% to 35% in Malawi, and 35% to 75% in Tanzania over the course of the periods in which the genomic data were collected. This is not incorporated into the analysis approach (e.g. by allowing parameter values to vary over time), and it is therefore unclear what the estimates obtained represent, or if they can be generalized to present day settings with higher ART coverage.

We agree that ART could have a considerable impact on *Mtb* infectiousness. We do not have individual-level ART data available, which was an important motivation for the CD4+ T-cell count analysis. CD4+ counts are expected to reflect the stage of HIV infection as well as whether a patient is on ART, and are therefore potentially more directly linked to the infectiousness of TB patients. When stratifying the population by CD4+ cell count instead of HIV infection status, we observed that the effect of low CD4+ counts on *Mtb* transmission is similar to the effect of HIV infection, indirectly suggesting that incorporating individual-level ART data, if available, would not have a strong impact on our results.

Incorporating a time-dependent, population-level effect of ART using ART coverage data is computationally challenging because of the increasing model complexity. Furthermore, our current estimates reflect an average for all HIV patients irrespective of ART, implying that patients on ART are currently potentially 'diluting' the HIV effect on *Mtb* transmission, and an even stronger effect can be expected when taking ART into account.

The prior distributions around the relative progression risk for PLHIV are unrealistic – it certainty shouldn't include numbers below 1, and there are plenty of data sources to provide a more reasonable prior. There is also little to support a prior on the relative R0 that goes above 1.

Although there is indeed some prior knowledge on the relative progression risk, the applied prior distributions on the relative progression risk and relative R_e put equal prior weights on values (i.e., multiplicative effects) above and below 1 to avoid any bias of our results. While these priors do put very low weight on extreme, unrealistic values, they are uninformative in terms of the direction of HIV

effects. The advantage of this choice is that we can consider the relative progression risk estimates as a 'positive control' in our results, increasing our confidence in the model.

The posterior distribution for South Africa for the relative infectious period of HIV+ TB falls almost entirely above 1, and has a median of around 3. This lacks face validity – prevalence to notification ratios are 53% lower for PLHIV than for HIV- people (doi:10.1111/tmi.13485). It is also in contrast to the results for the other three settings.

As mentioned above, the estimates for the relative infectious period are rather uncertain and strongly depend on the healthcare setting. These estimates are therefore not expected to be similar across different countries. Furthermore, the prevalence-to-notification ratio ignores the duration of infection prior to care-seeking and is therefore not equal to the total infectious period (from infection to recovery/death) used in our analyses.

Table S4 shows that only (very) small proportion of people with notified TB had samples included in the analysis. There will be biases in who made it into the dataset, which will vary between settings. This includes (but is not limited to) biases driven by study design – e.g. people diagnosed in hospital only in Tanzania. The large proportion of people with TB who are never diagnosed will also not be represented in the samples, and treatment coverage is likely to vary by HIV status. The implications of these biases on the results should be discussed.

It is true that TB patients who never get diagnosed are not represented in the datasets, and we agree that we cannot exclude potential sampling biases. At each sampling location, TB cases were identified through passive case finding (we now also explicitly mentioned this in the Materials and Methods section, Lines 279-280), although there might be other - potentially study-specific - sources of bias. To assess the effect of potential HIV-related sampling biases, we ran additional sensitivity analyses under 'extreme' sampling scenarios, showing that our qualitative conclusions remain unchanged (see Supplementary Figure S12 in the revised manuscript and our reply to the next comment).

Only sequences from patients with known HIV status were retained. What proportion of all sequences was this? What would be the potential impact of the probable differential missingness by HIV status? I did not understand the 'Randomization of HIV status' sensitivity analysis at all, I am not sure if that was designed to address this?

Information about HIV status was available for 3,601/3,638 sequences. We have specified missingness per location in the Materials and Methods section (Lines 294-295, Lines 302-303).

To assess the effect of differential missingness by HIV status, we conducted new analyses assuming either a twofold undersampling (e.g., due to reduced culturability) of *Mtb* from HIV-positive patients, or a twofold oversampling (e.g., due to better access to diagnosis) of HIV-positive patients. These two scenarios can be considered as 'extremes', and in both cases the qualitative conclusions remain unchanged (see Supplementary Figure S12 in the revised manuscript).

The randomization procedure was not designed to address this, but rather to evaluate where the signal in the data comes from and to identify potential model specification biases. By randomly assigning an HIV status to each patient in the dataset and conducting the analysis on this artificial dataset, the effects of HIV on *Mtb* transmission are expected to be lost. We found that this is indeed the case, but only when the HIV prevalence in the dataset is assumed to be similar to the prevalence in the general population. We can learn two things from this: 1) the increased HIV prevalence in TB patients is an important part of the data, driving our results to a large extent, and 2) when completely removing any information about HIV status (including prevalence) from the dataset, we no longer observe an effect of HIV on *Mtb* transmission, indicating that our results are not biased by our model assumptions.

Reviewer #2: 1- Can the authors describe the sampling of WGS in the results in more detail? were the patients with TB all comers to clinic? were all patients recruited in those studies sequenced? or were some missing. Was the expected missing rate of culture and sequencing preferrentially higher for HIV

(that is what I expect given patients with HIV are less likely to be microbiologically confirmed). What is the expected effect of undersampled patients with TB on their analysis results?

We now specified in the Materials and Methods section that all cases were identified through passive case finding (Lines 279-280). Additional details about the sampling can be found in the corresponding publications (Goig et al., 2023; Zwyer et al., 2023; Guerra-Assunção et al., 2015; Guwatudde et al., 2003; Wampande et al., 2013).

We agree that it is important to assess the effects of potential HIV-related sampling biases (see previous response to reviewer #1). There could indeed be undersampling of *Mtb* genomes from HIV-infected TB patients due to lower culture positivity. In South Africa and Tanzania, a slightly increased risk of missing sequence data was observed for HIV-positive patients (Goig et al.,2023; Zwyer et al., 2023). In Malawi, no such effect was observed (Guerra-Assunção et al., 2015). To be conservative, we tested the effect of a twofold lower sampling proportion for HIV-positive compared to HIV-negative patients.

Another important factor in the sampling process is the probability of HIV-positive TB patients to come to the clinic and get diagnosed. Because of their increased disease progression risk, there could also be a slight oversampling of HIV-positive TB patients. We therefore also investigated the effect of a twofold lower sampling proportion for HIV-negative compared to HIV-positive cases.

In both of the above cases, the qualitative conclusions remain unchanged: the relative reproductive number of HIV-positive patients remains below 1 while the relative progression risk remains above 1 (see Supplementary Figure S12 in the revised manuscript). The undersampling case which - as suggested by the reviewer – corresponds to the more likely scenario, results in stronger HIV effects, while the oversampling case results in weaker effects on *Mtb* transmission.

2- The authors describe that the date information was largely sufficient (over genomic and date data) to infer a lower transmission rate for HIV than for non-HIV index cases. The authors attribute this to dispersal of HIV/TB cases over the sampled TB phylogeny. Perhaps a related explanation is that the date and genetic data is highly correlated, can the authors run the model without dates and assess if the conclusions hold? related to this is whether there is sufficient temporal signal in the trees observed, and if they can elaborate on if molecular clock rates are being estimated or used from the literature. If they are being estimated how does the tree topology change with and without the dates?

As phylodynamic analyses by definition require a dated phylogenetic tree, the isolation dates are an essential part of the model and cannot be left out. When only a few dates are missing, they can in principle be inferred based on the sequences (tip dating). However, tip dating on all the tips is unlikely to be successful, especially when the temporal signal in the sequences is limited.

That said, we can indeed expect a correlation between the sequences and the isolation dates, provided that the sequences contain a strong clock signal. Since the sampling periods were relatively short, the clock signal is rather weak for the datasets used in this study. For this reason, we estimated the clock rate while assuming a strict clock model and setting a relatively strong prior (Lognormal(-16,1)) on the clock rate (also see Table S2 and Materials and Methods). This prior distribution is based on literature (Menardo et al., 2019), and reflects some prior uncertainty. Because of this uncertainty and the limited temporal signal in the data, we ran sensitivity analyses with the clock rate fixed to either 10^{-8} or 10^{-7} substitutions per site per year. These sensitivity analyses resulted in similar estimates for the HIV effects (Supplementary Figure S10), which is not surprising as the signal for these parameters mainly comes from the isolation dates (determining the number of cases over time) and HIV prevalence.

3- THe authors should temper down the conclusions they draw about the lack of association between CD4 count and transmissibility, especially given the lack of ART data. Can they also clarify the total sample size that supported the CD4 analysis? Also can they specifically assess the reproductive number among index cases with HIV and low CD4 count vs as a group the (non-HIV patients + HIV with high CD4)?

We now mentioned the number of patients for which CD4+ T-cell counts were available in the Materials and Methods section (Lines 294-295, Lines 315-316) as well as in the caption of Supplementary Figure S15.

We did not intend to state a lack of association between CD4+ T-cell counts and transmissibility, but instead stated that "CD4+ T-cell count is not a better predictor of *Mtb* transmissibility than HIV infection status alone" (Abstract) and that "the CD4+ T-cell count can be used as a predictor of *Mtb* transmission, but that, within the context of our analyses, it is not more informative than the HIV status" (Lines 178-180).

We now also rephrased this in the Discussion to further clarify this (Lines 221-224): "As the CD4+ T-cell count classification (low/high) for these patients largely overlapped with their HIV infection status (S15 Fig.), the observed effects of low CD4+ T-cell counts were largely similar to the effects of HIV co-infection."

Given the complete overlap between the low CD4+ count group and the HIV-positive group of patients (see Supplementary Figure S15), the comparison low vs. high CD4+ count already corresponds to the comparison suggested by the reviewer (HIV-pos and low CD4 vs. HIV-neg + HIV-pos with high CD4).

Part III – Minor Issues: Editorial and Data Presentation Modifications
Please use this section for editorial suggestions as well as relatively minor modifications of existing data that would enhance clarity.

Reviewer #1: "The TB notification rate in Temeke in 2020 was 3,994 cases per year" – that is an absolute number of cases not a rate, and hard to interpret without knowing the population size

We rephrased this sentence (Lines 303-304): "In 2020, 3,994 TB cases were notified in Temeke".

While TB notification rates are indeed usually given per 100,000 population, we were interested in the absolute number of cases reported during the sampling period, in order to estimate the sampling proportion. Hence, the population size is less relevant in this context.

"ART only recently became widely accessible to HIV patients in South Africa and Uganda" – this is not true, e.g. estimated ART coverage in South Africa reached 50% in 2014.

We would argue that an ART coverage of 50% in 2014 implies that the majority of HIV patients (i.e., since the time of HIV introduction, which is what is modelled here) did not have access to ART, hence the overall effect of ART can be expected to be limited.

"As we could only investigate the overall risk of developing active TB 188 disease after contact with a TB patient, it remains unclear, based on our data, whether 189 HIV also affects the risk of Mtb infection." – it is the overall risk of developing TB, not risk after contact (if it is indeed the latter, then that needs to be made much clearer in the methods")

Our parameter estimate does reflect the relative risk of developing TB after contact with an infectious individual, assuming that HIV-negative and HIV-positive individuals have similar contact rates. The HIV prevalence parameter (p_{HIV}) is used to account for different probabilities of contact with HIV-negative and HIV-positive people.

We have now clarified this in the Results and Materials and Methods sections:

Lines 76-78: "…a parameter for the multiplicative effect of HIV co-infection on the risk of getting diagnosed for active TB disease after contact with an infectious individual (f_2)…"

Lines 352-353: "…the multiplicative effect of HIV co-infection on the risk of TB disease development upon contact with an infectious individual (f_2)…"

Be careful about referring to people as ‘TB patients’ when you are talking about infectiousness – once they are diagnosed, become patients, and start treatment their infectiousness is greatly reduced – it is the period before they become patients where the vast majority of transmission is thought to occur

The infectious period in the model is indeed defined as the total period of infectiousness, and for most patients mainly occurs before diagnosis and treatment initiation. We agree that the term ‘patients’ is not strictly correct for people living with TB who are not yet on treatment. We therefore replaced it with ‘cases’ or ‘(infected) individuals’ throughout the manuscript, wherever information about diagnosis is lacking.

It should be made clear that the study is on pulmonary TB only

We have now specified for each study whether it included only pulmonary TB cases (Tanzania and Uganda) or both pulmonary and extrapulmonary TB cases (Malawi and South Africa) (Lines 283, 291, 299, 305).

The link to the code doesn’t work

The code is now accessible on GitHub. We also added all metadata as Supplementary Table.

Reviewer #2: 4- minor comment, the second paragraph is not informative, I wouldn't descirbe the association between HIV and active TB as a "finding", and suggest deleting this paragraph.

We agree that this is not a novel finding and have rephrased some sentences to clarify this:

Lines 104-108: “These results suggest that HIV-positive individuals have a 4- to 11-fold increased risk of developing active TB upon exposure, which is in accordance with the increased TB incidence rate observed in HIV patients [9, 10, 12, 13], and can thus be considered as a 'positive control' in our analyses.”

Lines 185-188: “Our phylodynamic analyses support previous studies showing that HIV/*Mtb* co-infected individuals are at high risk of developing active TB disease compared to HIV-negative *Mtb*-infected individuals. This observation serves as a ‘positive control’ that supports the validity of our method.”

Lines 192-193: “The finding from this and previous studies that HIV co-infection is a strong risk factor for developing active TB disease upon exposure...”

PLOS authors have the option to publish the peer review history of their article (what does this mean?). If published, this will include your full peer review and any attached files.

If you choose “no”, your identity will remain anonymous but your review may still be made public.

Do you want your identity to be public for this peer review? For information about this choice, including consent withdrawal, please see our Privacy Policy.

Reviewer #1: No

Reviewer #2: **Yes:** Maha Farhat
